# Acute management of unstable angina and non-ST segment elevation myocardial infarction

**DOI:** 10.1590/S1679-45082015RW3172

**Published:** 2015

**Authors:** Fernando Morita Fernandes Silva, Antonio Eduardo Pereira Pesaro, Marcelo Franken, Mauricio Wajngarten

**Affiliations:** 1 Hospital Israelita Albert Einstein, São Paulo, SP, Brazil.; 2 Faculdade de Medicina, Universidade de São Paulo, São Paulo, SP, Brazil.

**Keywords:** Angina, unstable, Myocardial infarction, Chest pain, Myocardial ischemia/drug therapy

## Abstract

Non-ST segment elevation coronary syndrome usually results from instability of an atherosclerotic plaque, with subsequent activation of platelets and several coagulation factors. Its treatment aims to reduce the ischemic pain, limiting myocardial damage and decreasing mortality. Several antiplatelet and anticoagulation agents have been proven useful, and new drugs have been added to the therapeutic armamentarium in the search for higher anti-ischemic efficacy and lower bleeding rates. Despite the advances, the mortality, infarction and readmission rates remain high.

## INTRODUCTION

The concept of acute coronary syndrome (ACS) encompasses different clinical presentations resulting from myocardial ischemia and includes unstable angina (UA), non-ST segment elevation myocardial infarction (NSTEMI) and ST-elevation myocardial infarction (STEMI). Ischemic heart disease is currently the major cause of mortality in Brazil and worldwide.^1^

Among the non-ST segment elevation acute coronary syndromes (NSTEACS) are UA and NSTEMI. Differentiation is primarily based on whether the ischemia is severe enough to cause myocardial damage and release markers of myocardial injury (troponins). However, the introduction of high-sensitivity troponins considerably reduced the prevalence of UA and considerably increased that of NSTEMI.

Non-ST segment elevation acute coronary syndromes presentation is heterogeneous, with different risk levels in terms of death, infarction and recurrence of infarction. Correct stratification of the death/reinfarction risk; early introduction of broad antithrombotic therapy, using two or three antiplatelet agents and one anticoagulant; and definition of the coronary functional/anatomical stratification method, whether invasive or noninvasive, are necessary for each patient.

The objective of this review was to provide a concise approach to the aspects that are currently more relevant in the treatment of NSTEACS.

## EARLY STRATIFICATION OF THE DEATH/INFARCTION RISK

Several clinical markers are associated with the risk of an unfavorable outcome in patients with NSTEACS, such as advanced age, diabetes, kidney failure, prolonged chest pain at rest, hypotension, tachycardia, and heart failure. However, the quantitative assessment using death/infarction risk scores is also a useful tool for the decision-making process.^2,3^

Some scores have been developed from different populations to estimate the ischemic (death, infarction, and recurrent ischemia) and bleeding risks. The Global Registry of Acute Coronary Events (GRACE),^2^ Thrombolysis in Myocardial Infarction (TIMI)^3^ and Braunwald^4^scores are those more frequently used worldwide. The first one is more complex and requires a software for the score calculation. Alternatively, the website http://www.outcomes.org/grace can be used for the estimate. It provides a good risk discrimination at hospital admission and discharge. The TIMI score is simpler to use, but its accuracy seems to be lower,^5^ possibly for not using hemodynamic data such as systolic pressure, heart rate and Killip classification.

Although, among the three, Braunwald’s classification is the easiest for bedside use, the introduction of high-sensitivity troponins may reduce this score’s sensitivity, since one positive troponin test is enough for the patient to be considered as having a high risk. Nonetheless, the score is still an option recommended for early stratification.

## INITIAL ANTI-ISCHEMIC THERAPY

After the diagnosis of NSTEACS is made, the initial therapy should address the following aspects: pain relief, early risk stratification, hemodynamic assessment, anticoagulation and anti-thrombotic therapy, invasive or conservative strategy, and monitoring and early treatment of arrhythmias. Resting and continuous electrocardiographic monitoring are recommended for all patients with NSTEACS during the initial in-hospital phase.^6^

## OXYGEN

Patients with oxygen saturation <90%, dyspnea or high risk for hypoxemia shoud be provided with supplemental oxygen.^6^ There is no evidence for its use in eupneic patients with no hypoxemia, and there is a remote risk of inducing hyperoxia and vasoconstriction.^7^

A meta-analysis published in Cochrane, assessing the routine use of oxygen in 430 patients with myocardial infarction, did not show any beneficial effect.^8^ In the recent AVOID (Air Verses Oxygen In myocarDial infarction) study,^9^ which included 441 patients with STEMI, the routine use of oxygen in non-hypoxemic patients was associated with recurrent ischemia, arrhythmias and larger infarct sizes at the end of 6 months.

## NITRATES

The use of nitrates in NSTEACS is mainly based on pathophysiological aspects and on the clinical experience. This class of drugs leads to vasodilatation in the coronary and peripheral circulation. It reduces the preload, the left ventricular end-diastolic volume, and, consequently, the myocardial oxygen consumption.

Although in previous studies there is no evidence of mortality reduction with the use of nitrates, these are still first-choice medications in patients with ischemic or congestive symptoms.^10^ They can cause headache and postural hypotension, which may be reverted by dose reduction and analgesics. They should be avoided in patients with hypotension, right ventricular infarction, and recent use (24 to 48 hours) of phosphodiesterase inhibitors (sildenafil, vardenafil or tadalafil).

## MORPHINE

When the anginal pain persists despite the use of nitrate, morphine may be used. In addition to its potent analgesic effect, the vasodilation action helps relieve pain and reduce blood pressure and the congestive symptoms.

Intravenous morphine sulfate may be used to control pain and anxiety, at doses from 2 to 4mg, repeated at 5 to 15-minute intervals.

## BETA-BLOCKERS

This class of drugs reduces myocardial oxygen consumption by decreasing heart rate, myocardial contractility and blood pressure. It prolongs diastole and increases coronary perfusion. It reduces the release of renin, angiotensin II and aldosterone by blocking beta-1 receptors in the renal juxtaglomerular cells, in addition to having antiarrhythmic effects, with a reduction in the risk of ventricular fibrillation.^11^

The clinical studies that corroborate the use of beta-blockers involved patients with non-specified infarction, however with a much higher proportion of individuals with STEMI. A meta-analysis showed a decrease by 23% in mortality (95% confidence interval – 95% CI: 15-31%) when they were used for a prolonged time after ACS.^12^ There are no randomized studies specifically conducted in a population with NSTEMI. However, observational evidences do not suggest different outcomes.^13^

Oral beta-blockers are indicated in all patients with NSTEACS for whom there are no contraindications such as active bronchospasm; hemodynamic instability; severe bradycardia; recent use of cocaine; atrioventricular blocks greater than first degree; and decompensated heart failure. In patients with compensated ventricular dysfunction, they must be used with care. Cardioselective beta-blockers (metoprolol or atenolol) are preferable because they act predominantly on beta-1 receptors and show a lower risk of bronchospasm at low doses.

The COMMIT/CCS2 (Clopidogrel and Metoprolol in Myocardial Infarction Trial/Second Chinese Cardiac Study),^14^which involved 45,852 patients with ACS (93% with STEMI), used intravenous metoprolol in an aggressive protocol (up to 15mg intravenously, followed by 200mg orally per day). There was a reduction in the risk of reinfarction and ventricular fibrillation, but an increase in the risk of development of cardiogenic shock. The use of intravenous beta-blockers should be avoided in patients with a higher possibility of developing cardiogenic shock (patients with advanced age, tachycardia, moderate to severe systolic dysfunction and hypotension).

## STATINS

All patients with ACS should receive statins at an intensive and early regimen, regardless of their low-density lipoprotein levels.^15^ The drug suggested is atorvastatin 80mg/day in comparison to other statin regimens, based on studies on ACS.^16^ Alternatively, rosuvastatin 20 to 40mg/day may be used.^15^

## ANTIPLATELET AGENTS

In the last decade, multiple antiplatelet therapy has been considered key for the successful treatment of ACS. Platelet activation and aggregation occurs via different pathways, therefore requiring that antagonization using antiplatelet agents include all pathways involved. Thus, the current antiplatelet therapy is made using two or three drugs combined^17^(Chart 1).

**Chart 1:** Antithrombotic medications

Drug	Dose	Time of use	Contraindications	Side effects	Renal function adjustment	Observation
ASA	Oral loading dose: 200mg	Lifelong	Bleeding	Anaphylaxis Bleeding Gastric ulcer	No	
	Maintenance: 100mg, once a day					

Ticagrelor	Oral loading dose: 180mg Maintenance: 90mg, twice a day	1 year	Sinus node disease	Ventricular pause (6%)	No	Keep loading dose if previous clopidogrel
			Second and third degree atrioventricular block	Dyspnea (13%)		
				Hyperuricemia (>10%)		
			Bleeding	Bleeding		

Prasugrel	Oral loading dose: 60mg	1 year	Age >75 years	Bleeding	No	
	Maintenance: 10mg, once a day		Weight <60kg			
			Previous stroke			
			Bleeding			

Abciximab	Intravenous loading dose: 0.25mg/kg	12 hours	Stroke <2 years	Bleeding		
	Maintenance: 0.125mcg/kg/minute (maximum 10mcg/minute)		Surgery/trauma <2 months			
			Cerebral neoplasia			
			Liver disease			
			Dialysis			
			Thrombocytopenia			
			Bleeding			

ASA: acetylsalicylic acid.

### Acetylsalicylic acid

Acetylsalicylic acid (ASA) exerts its action by acetylating cyclooxygenase-1 (COX-1), irreversibly inhibiting the enzyme responsible for the conversion of arachidonic acid into thromboxane A2. The reduced thromboxane A2 activity inhibits platelet activation, degranulation and aggregation. The action of ASA in ACS was evaluated in several classic randomized studies.^18^ In all of them, ASA was able to reduce the relative risk of death or reinfarction by up to 64%. This drug should be introduced immediately after the diagnosis in all patients, at an initial loading dose of 162 to 325mg, followed by a daily maintenance dose of 100mg, indefinitely. It should only be avoided in patients with history of allergy to the drug, bleeding peptic ulcer or active bleeding. Although ASA is a mandatory drug in ACS, high rates of residual platelet hyperactivity (up to 30%) have been observed in patients on ASA.

### Thienopyridines (clopidogrel, ticagrelor and prasugrel)

Thienopyridines exert their action by inhibiting the adenosine diphosphate (ADP) receptor on the platelet surface. The CURE (Clopidogrel in Unstable Angina to Prevent Recurrent Events) trial^19^ analyzed the effect of clopidogrel combined with ASA in NSTEACS. A total of 12,562 patients within the first 24 hours of the onset of symptoms were randomized to receive clopidogrel *versus* placebo combined with ASA for 3 to 12 months. The combination reduced the risk of composite cardiovascular events (acute myocardial infarction – AMI, cardiovascular death, and stroke) by approximately 20%. The reduction in the relative risk reached 30% in patients undergoing angioplasty with stent implantation. This beneficial effect was observed in low-, medium- and high-risk patients.

More recently, shorter-acting, more potent ADP inhibitors proved superior to clopidogrel. Prasugrel was superior to clopidogrel in the TRITON study,^20^ which assessed STEMI and NSTEMI patients, showing lower rates of composite events of reinfarction, stent thrombosis and death (RRR=19%, NNT=46). However, bleeding was more frequent with an absolute increase in the prasugrel group, in the subgroups of patients with previous stroke, >75 years of age or low weight (<60kg). Ticagrelor was also superior to clopidogrel in the PLATO (Platelet Inhibition and Patient Outcomes) study,^21^ whose sample comprised patients with STEMI and NSTEMI, whether using an invasive or conservative approach. There was a reduction in the risk of reinfarction, stent thrombosis and death (RRR=16%; NNT=54), with a slight absolute increase in the bleeding risk.

Thus, the new ADP inhibitors may be considered first-line options. Ticagrelor may be used from patient admission – including those who were on clopidogrel, which can be replaced by ticagrelor. Prasugrel should only be administered in the cases for which angioplasty will certainly be performed, in patients not previously on clopidogrel. When several high risk factors for hemorrhage are present (very advanced age, women, low weight, use of warfarin, and kidney failure), clopidogrel is a safer and more feasible option.

Considering the results of the TRITON and PLATO studies, glycoprotein (GP) IIb/IIIa inhibitors became second-line options that can be used in a triple therapy, in combination with ASA and an ADP inhibitor in selected cases of patients with ACS undergoing angioplasty (high thrombotic load, no-reflow, and distal embolization).

## ANTICOAGULANTS

Anticoagulants are drugs that inhibit thrombin generation and/or activity. The use of anticoagulants in ACS is a subject of active investigation. It is hard to obtain definitive conclusions on the best anticoagulation strategy because of different treatment times, uncertainty regarding equipotent anticoagulant doses, and different antiplatelet drugs used in the studies. Several anticoagulants have been tested, but currently four drugs are available for the use in NSTEACS, namely: unfractionated heparin, enoxaparin, fondaparinux and bivalirudin (Chart 2).

**Chart 2:** Anticoagulation therapy

Drug	Dose	Time of use	Contraindication	Side effects	Adjustments	Observation
Unfractionated heparin	IV loading dose: 60IU/kg maximum 5,000IU	48 hours or discontinue after angioplasty	Active bleeding Thrombocytopenia	Thrombocytopenia Bleeding	According to PTT	Antidote: protamine
	IV Maintenance:12IU/kg/h (maximum 1,000IU/h)			Increased transaminase levels		
	Keep PTT between 50 and 70 seconds (ratio 1.5 to 2.0)					
	In angioplasty keep ACT 200-300 seconds					

Enoxaparin	Subcutaneous 1mg/kg and every 12 hours up to 100kg	8 days or discontinue after angioplasty	Active bleeding Thrombocytopenia	Thrombocytopenia Bleeding	50% dose reduction dose in AKF/CKF	Monitor anti-Xa in obese, elderly, AKF/CKF patients
	For angioplasty, if last dose between 8-12 hours or if only one enoxaparin dose administered: 0.3mg/kg IV			Increased transaminase levels	Optional: 25% reduction in the very elderly	Antidote: protamine (partial effect)

Fondaparinux	Subcutaneous 2.5mg once a day	8 days or discontinue after angioplasty	Active bleeding	Bleeding, anemia	No adjustments	Combine with heparin if angioplasty
			Creatinine clearance <20mL/min			

V: intravenous; PTT: partial thromboplastin time; ACT: activated coagulation time; AKF: acute kidney failure; CKF: chronic kidney failure.

### Unfractionated heparin

Unfractionated heparin (UFH) is a heterogeneous mixture of polysaccharides with molecular weights ranging between 2,000 and 30,000 daltons. It exerts its effect by linking to antithrombin and potentiating its action. It has a narrow therapeutic range and requires frequent monitoring by means of partial thromboplastin time.

Anticoagulation with UFH has been the cornerstone of therapy for patients with UA/NSTEMI based on several randomized studies which found lower death or reinfarction rates with the use of UFH combined with ASA in relation to ASA alone.^22,23^

Unfractionated heparin should be initially administered as an intravenous bolus of 60 units per kg up to a maximum dose of 5,000IU, followed by continuous infusion of 12IU/kg/hour, up to a maximum dose of 1,000IU/hour. Dose titration should be based on the partial thromboplastin time, targeting between 50 and 70 seconds. Daily determination of hemoglobin, hematocrit and platelet levels are recommended.

### Enoxaparin

Low-molecular-weight heparins (LMWH) are obtained from UFH depolymerization and selection of those with lower molecular weights (between 2,000 and 10,000 daltons). They show better subcutaneous absorption, lower protein binding, less platelet activation and a more predictable and reproducible effect. Anticoagulation control or dose titration are usually unnecessary. Only in patients with kidney failure or, occasionally, in obese and elderly patients, control of LMWH action by anti-Xa activity determination is recommended.

The ESSENCE^24^and TIMI 11B^25^studies, which compared enoxaparin with UFH in combination with two antiplatelet agents in patients undergoing an initial conservative approach, suggested an anti-ischemic beneficial effect with enoxaparin.

In the SYNERGY study,^26^ which involved 10,027 patients who received a current treatment strategy with early angiography and the use of GP IIb/IIIa platelet inhibitor, enoxaparin and UFH, similar anti-ischemic results were obtained. The bleeding rate was higher with enoxaparin using the TIMI criteria (9.1% *versus* 7.6%; p=0.008), however without statistically significant difference using the GUSTO criteria or greater need for transfusion. Those using a heparin formulation and later the other, prior to coronary cineangiography, showed higher rates of bleeding and of the composite endpoint of death or infarction.

Patients with high-risk UA/NSTEMI undergoing an early invasive strategy, including those using GP IIa/IIIb inhibitors, may receive enoxaparin or UFH. However, once one of them is chosen, maintenance of the same drug until treatment completion is recommended.

### Fondaparinux

Fondaparinux is a synthetic pentasaccharyde analogous to the antithrombin binding site present in heparin molecules. It exerts its action by neutralizing the Xa factor thus preventing thrombin generation. It has an excellent bioavailability after subcutaneous injection, and a plasma half-life of 17 hours, which permits its administration once a day. It has renal elimination exclusively and should not be used in patients with clearance <20mL/minute. No definitive case of fondaparinux-induced autoimmune thrombocytopenia has been reported.^27^

In the OASIS-5 study,^28^ 20,078 patients with NSTEACS were randomized to receive either subcutaneous fondaparinux 2.5mg once a day or enoxaparin 1mg/kg twice a day, for 8 days or until hospital discharge. The group using fondaparinux showed a reduction in the ischemic events risk similar to that of the group using enoxaparin, and there was a substantial reduction in the major bleeding (2.2 *versus *4.1%; p<0.001) and fatal bleeding rates (7 *versus *22%; p=0.005), respectively.

Fondaparinux was associated with a lower 30-day (2.9% *versus *3.5%; p=0.02) and 180-day mortality rate (5.8% *versus *6.5%; p=0.05). However, in patients undergoing percutaneous revascularization, there was more catheter-related thrombosis in the fondaparinux group (0.9% *versus* 0.4%; p=0.001), which resulted in the recommendation of the use of UFH or bivalirudin in patients on fondaparinux undergoing angioplasty. Despite this excellent result with fondaparinux, we should mention that, in the enoxaparin group, many patients received an additional UFH dose at the moment of angioplasty, a measure that is currently contraindicated due to the increase in the bleeding risk.^26^

In sum, fondaparinux proved to be a safer option for patients with ACS. Patients with NSTEACS undergoing conservative treatment benefit from a lower bleeding risk. If angioplasty is indicated, the combination of UFH during the procedure prevents catheter-related thrombosis, apparently without increasing the bleeding risk. It is an alternative in invasive approaches (<72 hours after admission). In those requiring an urgent/emergency invasive procedure (<2 hours after admission), fondaparinux is not recommended and, probably, UFH or bivalirudin are the best drugs in this situation.^27^

### Bivalirudin

Bivalirudin belongs to the group of direct thrombin inhibitors that bind to and inactivate one or more sites in the thrombin molecule. Bivalirudin is a synthetic polypeptide analogous to hirudin. Since it does not bind to plasma proteins, its anticoagulant effect is more predictable. Unlike heparins, it does not require a cofactor to act, and may inhibit clot-linked thrombin. Its half-life is of approximately 25 minutes in patients with normal renal function, and the coagulation parameters return to normal approximately 1 hour after discontinuation.

Bivalirudin was tested in patients with NSTEACS in the ACUITY study.^29^ A total of 13,819 individuals were randomized to three groups: UFH or enoxaparin in combination with GP IIb/IIIa inhibitor; bivalirudin in combination with GP IIb/IIIa inhibitor; or bivalirudin alone (9.1% of patients in the latter group received GP IIb/IIIa inhibitor). The incidence of composite endpoints of 30-day ischemia (death of any cause, myocardial infarction or unplanned revascularization) were similar in the three groups, but there was less major bleeding in the group that used bivalirudin alone in comparison to heparins plus GP IIb/IIIa inhibitors (3.0% *versus* 5.7%; p<0.001).

We can state that bivalirudin in combination with dual antiaggregation (or triple, in selected cases) has the same efficacy profile and lower bleeding rates than routine early heparin in triple antiaggregation therapy, but we do not have data to simply state that bivalirudin is superior to heparins in ACS. We do not recommend triple antiaggregation routinely in patients with NSTEACS.

**Figure 1:**
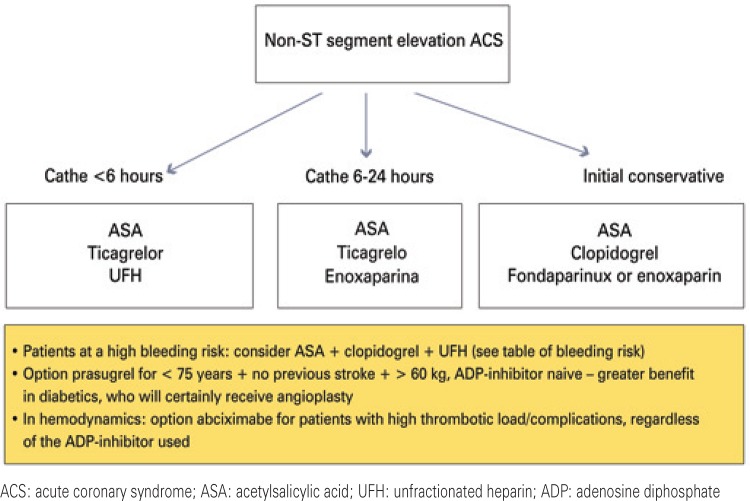
Flowchart of the antithrombotic therapy according to stratification, in non-ST segment elevation acute coronary syndrome in *Hospital Israelita Albert Einstein*

The choice of the combination of antiaggregant and anticoagulant involves patients’ characteristics, bleeding risk, drug availability, and the definition of either an invasive or a conservative strategy. Figure 1 shows a flowchart of the suggested antithrombotic treatment of NSTEACS, according to stratification, used in *Hospital IsraelitaAlbert Einstein*.

## CONSERVATIVE AND INVASIVE STRATEGIES

After the diagnosis of NSTEACS is made, in addition to drug therapy, the patients should undergo some type of stratification strategy using ancillary tests for the functional (noninvasive ischemia tests) or anatomical assessment of the coronaries (coronary cineangiography). Generally, the choice of the method depends on the patient’s risk, the presence of comorbidities, life expectancy, functional status, and the availability of stratification methods in each medical service. We can chose from immediate coronary cineangiography (within 2 hours of admission), invasive strategy (coronary cineangiography within 48 to 72 hours), and conservative strategy.

Immediate coronary cineangiography in NSTEACS is recommended in the following groups of unstable patients at a high risk of unfavorable outcome: recurrent or persistent angina, despite intensive medical treatment; hemodynamic instability; severe ventricular dysfunction and heart failure; sustained ventricular arrhythmia; and mechanical complications (acute mitral regurgitation and ventricular septal defect).

Invasive strategy (coronary cineangiography targeted at revascularization within 48 to 72 hours) may limit the extension of the infarct area and improve the prognosis of patients with NSTEACS at a moderate and high risk.^30^ We suggest the invasive strategy when the following characteristics are present, always considering the patients’ bleeding risk and functional status: TIMI score ≥3; GRACE score ≥108; troponin elevation; new-onset or presumably new-onset ST depression; ejection fraction <40%; coronary angioplasty in the past 6 months or previous coronary artery bypass grafting; and post-infarction angina.

In patients at a higher risk, such as those with GRACE score ≥140, the invasive strategy within the first 24 hours (14 hours, on average) was superior in the TIMACS (Timing of Intervention in Acute Coronary Syndrome) study^31^ when compared to the invasive strategy after 36 hours (50 hours, on average).

The conservative strategy consists of the performance of a noninvasive ischemia test, such as exercise test, pharmacological- (adenosine, dipyridamole, dobutamine) or exercise-stress myocardial scintigraphy, and dobutamine echocardiography after the event. Any of these methods is sensitive in the detection of ischemia and assessment of patient risk. The option is for the test that is more appropriate according to the availability in the medical service, patient’s physical conditions (ability to exercise), drug tolerance (patients with asthma should not use adenosine or dipyridamole) and baseline electrocardiogram (patients with bundle branch blocks, pacemakers or severe ventricular overload should undergo imaging studies).

In patients at a low risk (GRACE score ≤108 or TIMI ≤2) who do not present with any of the characteristics previously described for the indication of invasive strategy, the initial conservative option seems to be more appropriate.

## CONCLUSION

The treatment of non-ST segment elevation acute coronary syndromes is continuously evolving with the inclusion of new antiplatelet and anticoagulant drugs, which target at higher anti-ischemic efficacy and lower bleeding rates. The adequate therapeutic approach using evidence-based interventions, in association with effective preventive measures, may help decrease morbidity and mortality.
