# Supplementary material for: Acute management of unstable angina and non-ST segment elevation myocardial infarction
Source: Einstein (Sao Paulo). 2015 Jul-Sep;13(3):454–61. doi: 10.1590/S1679-45082015RW3172 (PMC4943796; doi:10.1590/S1679-45082015RW3172)
Supplement: Translation

# Translation: Tratamento atual da síndrome coronária aguda sem supradesnivelamento do segmento ST

**Authors:** Fernando Morita Fernandes Silva, Antonio Eduardo Pereira Pesaro, Marcelo Franken, Mauricio Wajngarten

**Affiliations:** 1 Hospital Israelita Albert Einstein, São Paulo, SP, Brasil.; 2 Faculdade de Medicina, Universidade de São Paulo, São Paulo, SP, Brasil.

**Keywords:** Angina instável, Infarto do miocárdio, Dor no peito, Isquemia miocárdica/quimioterapia

## Abstract

A síndrome coronária sem supradesnivelamento do ST geralmente resulta da instabilização de uma placa aterosclerótica, com subsequente ativação plaquetária e de diversos fatores de coagulação. O tratamento visa aliviar a dor isquêmica, limitar o dano miocárdico e diminuir a mortalidade. Diversos agentes antiagregantes e anticoagulantes provaram sua utilidade, e novas drogas passaram a compor o arsenal terapêutico, buscando maior eficácia anti-isquêmica e menores índices de sangramento. Apesar dos avanços, as taxas de mortalidade, infarto e reinternação ainda permanecem elevadas.

## INTRODUÇÃO

O conceito de síndrome coronária aguda (SCA) compreende diversas apresentações clínicas decorrentes de isquemia miocárdica, abrangendo a angina instável (AI), o infarto agudo do miocárdio sem supradesnivelamento de ST (IAMSS-ST) e o infarto agudo do miocárdio com supradesnivelamento de ST (IAMCS-ST). Atualmente, a doença cardíaca isquêmica é a principal causa de mortalidade no Brasil e no mundo.^1^

Entre as síndromes coronárias agudas sem supradesnivelamento do ST (SCASS-ST), estão incluídas a AI e o IAMSS-ST. A diferenciação ocorre primariamente se a isquemia é suficientemente severa para causar dano miocárdico e liberar marcadores de injúria miocárdica (troponinas). No entanto, o advento das troponinas ultrassensíveis reduziu consideravelmente a frequência de AI e elevou consideravelmente a de IAMSS-ST.

A SCASS-ST se apresenta de forma heterogênea com diferentes níveis de risco em termos de morte, infarto e recorrência do infarto. Para cada paciente, é necessário: estratificar corretamente o risco de morte/reinfarto; implementar precocemente o tratamento antitrombótico amplo, com dois ou três antiplaquetários e um agente anticoagulante; definir o método de estratificação funcional/anatômica coronária, de forma invasiva ou não invasiva.

Esta revisão pretendeu abordar, de forma concisa, os aspectos atuais mais relevantes do tratamento das SCASS-ST.

## ESTRATIFICAÇÃO PRECOCE DE RISCO DE MORTE/INFARTO

Diversos marcadores clínicos se associam ao risco de evolução desfavorável nos pacientes com SCASS-ST como: idade avançada, diabetes, insuficiência renal, dor precordial prolongada em repouso, hipotensão, taquicardia e insuficiência cardíaca. Porém a avaliação quantitativa por meio dos escores do risco de morte/infarto é também ferramenta útil para a tomada de decisões.^2,3^

Alguns escores foram desenvolvidos a partir de diferentes populações para estimar os riscos isquêmicos (morte, infarto e isquemia recorrente) e hemorrágicos. Os escores *Global Registry of Acute Coronary Events* (GRACE),^2^ *Thrombolysis in Myocardial Infarction* (TIMI)^3^e Braunwald^4^ são os mais usados globalmente. O primeiro é mais complexo e necessita da utilização de *software* para seu cálculo. Alternativamente, é possível utilizar o *site* http://www.outcomes.org/grace para sua estimativa. Permite boa discriminação de risco na admissão e alta hospitalar. O escore de TIMI é mais simples de utilizar, mas sua acurácia parece ser menor,^5^ possivelmente por não utilizar dados hemodinâmicos, como pressão sistólica, frequência cardíaca e classificação de Killip.

Apesar do escore de Braunwald ser o mais simples dos três para aplicação à beira do leito, o advento das troponinas ultrassensíveis pode reduzir a sensibilidade desse escore, já que basta uma troponina positiva para o paciente ser considerado de alto risco. Mesmo assim, o escore ainda é uma opção recomendada para estratificação precoce.

## TRATAMENTO ANTI-ISQUÊMICO INICIAL

Após o diagnóstico de SCASS-ST, o tratamento inicial deve abordar os seguintes aspectos: alívio da dor, estratificação precoce de risco, avaliação hemodinâmica, terapêutica antitrombótica e anticoagulante, estratégia invasiva ou conservadora, monitorização e tratamento precoce de arritmias. Repouso e monitorização eletrocardiográfica contínua são recomendados para todos o pacientes com SCASS-ST durantes a fase hospitalar inicial.^6^

## OXIGÊNIO

Oxigênio suplementar deve ser ofertado para os pacientes que apresentem saturação <90%, dispneia ou alto risco para hipoxemia.^6^ Não há evidência para seu uso em pacientes eupneicos sem hipoxemia e existe o risco remoto de indução de hiperóxia e vasoconstricção.^7^

Metanálise publicada na Cochrane, que avaliou o uso rotineiro de oxigênio em 430 pacientes com infarto do miocárdio, não evidenciou benefício.^8^No recente estudo AVOID (*Air Verses Oxygen In myocarDial infarction study*),^9^ que incluiu 441 pacientes com SCACS-ST, a utilização rotineira de oxigênio em pacientes não hipoxêmicos esteve associada a isquemia recorrente, arritmias e maiores infartos ao final de 6 meses.

## NITRATOS

A utilização de nitratos em SCASS-ST é baseada principalmente em aspectos fisiopatológicos e na experiência clínica. Essa classe de drogas causa vasodilatação em circulação coronária e periférica. Diminui a pré-carga, o volume diastólico final do ventrículo esquerdo e, consequentemente, o consumo miocárdico de oxigênio.

Apesar de não haver evidência em estudos prévios de redução de mortalidade com o uso de nitratos, ainda são medicações de primeira escolha em pacientes com sintomas isquêmicos ou congestivos.^10^ Podem causar cefaleia e hipotensão postural, as quais podem ser revertidas com diminuição da dose e analgésicos. Devem ser evitados em pacientes com hipotensão, infarto do ventrículo direito e uso recente (24 a 48 horas) de inibidores da fosfodiesterase (sildenafil, vardenafil ou tadalafil).

## MORFINA

Quando houver persistência de dor anginosa, apesar do uso de nitrato, morfina pode ser utilizada. Além do potente efeito analgésico, a ação vasodilatadora colabora com o alívio da dor, e a redução de pressão arterial e dos sintomas congestivos.

Sulfato de morfina endovenoso pode ser utilizado para controle de dor e ansiedade em doses de 2 a 4mg, repetindo-se em intervalos de 5 a 15 minutos.

## BETABLOQUEADORES

Essa classe de drogas diminui o consumo miocárdico de oxigênio por meio da diminuição da frequência cardíaca, da contratilidade miocárdica e da pressão arterial. Prolonga a diástole e aumenta a perfusão coronária. Reduz a liberação de renina, angiotensina II e aldosterona, bloqueando os receptores beta-1 nas células renais justaglomerulares, além de apresentar efeitos antiarrítmicos, com diminuição do risco de fibrilação ventricular.^11^

Os ensaios clínicos que embasam o uso de betabloqueadores envolveram pacientes com infarto não especificado, mas com proporção muito maior de indivíduos com IAMCS-ST. Metanálise demonstrou diminuição da mortalidade em 23% (intervalo de confiança de 95% − IC95%: 15-31%) quando utilizados por tempo prolongado após SCA.^12^ Não há estudos randomizados especificamente na população com IAMSS-ST, mas evidências observacionais não sugerem desfechos diferentes.^13^

Betabloqueadores orais são indicados em todos os pacientes com SCACS-ST que não tenham contraindicações, como broncoespasmo ativo, instabilidade hemodinâmica, bradicardia severa, uso recente de cocaína, bloqueios atrioventriculares maiores que de primeiro grau e insuficiência cardíaca descompensada. Em pacientes com disfunção ventricular compensada, devem ser usados com cautela. Utilizar preferencialmente os cardiosseletivos (metoprolol ou atenolol), que apresentam predomínio de ação sobre os receptores beta-1 e menor risco de broncoespasmo em baixas doses.

O estudo COMMIT/CCS2 (*Clopidogrel andMetoprolol in Myocardial Infarction Trial/Second ChineseCardiac Study*),^14^ que envolveu 45.852 pacientes com SCA (93% com SCACS-ST), utilizou metoprolol endovenoso em um protocolo agressivo (até 15mg endovenosas, seguidas de 200mg em formulação oral por dia). Houve diminuição do risco de reinfarto e fibrilação ventricular, mas aumento do risco de evolução para choque cardiogênico. Recomenda-se evitar o uso de betabloqueador endovenoso em pacientes com maior possibilidade de evolução para choque cardiogênico (idosos, taquicárdicos, disfunção sistólica moderada a importante, e hipotensos).

## ESTATINAS

Todos os pacientes com SCA devem receber estatinas em regime intensivo e com início precoce, independentemente dos valores de lipoproteína de baixa densidade.^15^ Sugere-se atorvastatina 80mg/dia em comparação com outros regimes de estatinas, com base em estudos em SCA.^16^ Alternativamente, pode-se utilizar rosuvastatina 20 a 40mg/dia.^15^

## ANTIPLAQUETÁRIOS

Na última década, a antiagregação plaquetária múltipla foi considerada fundamental para o sucesso do tratamento das SCA. A ativação e a agregação plaquetária ocorre por vias distintas, o que demanda que a antagonização por fármacos antiplaquetários contemple todas as vias envolvidas. Desse modo, o tratamento atual antiplaquetário é feito com dois ou três fármacos combinados^17^ (Quadro 1).

**Quadro 1:** Medicações antitrombóticas

Fármaco	Dose	Tempo de uso	Contraindicações	Efeitos colaterais	Ajuste função renal	Observação
AAS	Oral ataque: 200mg	Permanente	Hemorragia	Anafilaxia	Não	
	Manutenção: 100mg, uma vez ao dia			Hemorragia		
				Úlcera gástrica		
Ticagrelor	Oral ataque: 180mg	1 ano	Doença do nó sinusal	Pausa ventricular (6%)		
	Manutenção: 90mg, duas vezes dia		Bloqueio atrioventricular de segundo e terceiro graus	Dispneia (13%) Hiperuricemia (>10%) Hemorragia	Não	Manter ataque se clopidogrel previamente
			Hemorragia			
Prasugrel	Oral ataque: 60mg	1 ano	Idade >75 anos	Hemorragia	Não	
	Manutenção: 10mg, uma vez dia		Peso <60kg			
			Acidente vascular cerebral prévio			
			Hemorragia			
Abciximab	Endovenoso	12 horas	Acidente vascular cerebral <2 anos	Hemorragia		
	Ataque: 0,25mg/kg		Cirurgia/trauma <2 meses			
	Manutenção: 0,125mcg/kg/minuto (máximo 10mcg/minuto)		Neoplasia cerebral			
			Hepatopatia			
			Diálise			
			Trombocitopenia			
			Hemorragia			

AAS: ácido acetilsalicílico.

### Ácido acetilsalicílico

O ácido acetilsalicílico (AAS) exerce sua ação pela acetilação da ciclo-oxigenase-1 (COX-1) inibindo, irreversivelmente, a enzima responsável pela conversão do ácido araquidônico em tromboxano A2. A redução da atividade do tromboxano A2 inibe a ativação, a degranulação e a agregação plaquetária. A ação do AAS nas SCA foi avaliada em diversos estudos randomizados clássicos.^18^ Em todos eles, o AAS foi capaz de reduzir o risco relativo de morte ou reinfarto em até 64%. Essa medicação deve ser introduzida imediatamente após o diagnóstico em todos os pacientes, na dose inicial de ataque de 162 a 325mg, seguida de dose de manutenção diária de 100mg, e mantida indefinidamente. Deve ser evitada apenas em pacientes com antecedente de alergia ao fármaco, úlcera péptica hemorrágica ou sangramento ativo. Apesar do AAS ser uma medicação obrigatória nas SCA, altas taxas hiperatividade plaquetária residual (até 30%) foram observadas em pacientes usuários de AAS.

### Tienopiridínicos (clopidogrel, ticagrelor e prasugrel)

Os tienopiridínicos exercem seu efeito inibindo o receptor da adenosina difosfato (ADP) na superfície plaquetária. O estudo CURE (*Clopidogrel in Unstable Angina to Prevent Recurrent Events Trial*)^19^analisou o efeito do clopidogrel associado ao AAS em SCASS-ST. Um total de 12.562 pacientes, nas primeiras 24 horas do início dos sintomas, foi randomizado para receber clopidogrel *versus* placebo, associados ao AAS, por 3 a 12 meses. A associação reduziu em cerca de 20% o risco de eventos cardiovasculares combinados (infarto agudo do miocárdio − IAM, morte cardiovascular e acidente vascular cerebral − AVC). A redução de risco relativo chegou a 30% nos pacientes submetidos a angioplastia com implante de *stents*. O benefício ocorreu em pacientes de baixo, médio e alto risco.

Mais recentemente, inibidores de ADP com ação mais rápida e maior potência mostraram-se superiores ao clopidogrel. O prasugrel foi superior ao clopidogrel no estudo TRITON,^20^ avaliando pacientes pós-IAM com e sem supradesnivelamento de ST, com menores taxas de eventos combinados de reinfarto, trombose de *stent* e óbito (RRR=19%, NNT=46). No entanto, os sangramentos foram mais frequentes com incremento absoluto no grupo prasugrel nos subgrupos de pacientes com AVC prévio, >75 anos ou com baixo peso (<60kg). O ticagrelor também foi superior ao clopidogrel no estudo PLATO (*Platelet Inhibition and Patient Outcomes*),^21^ cuja amostra era de pacientes com IAM com e sem supradesnivelamento de ST, com estratégia invasiva ou conservadora. Houve redução do risco de reinfarto, trombose de *stent* e óbito (RRR=16%; NNT=54), com incremento absoluto discreto do risco de sangramento.

Desse modo, os novos inibidores de ADP podem ser considerados opções de primeira linha. Ticagrelor pode ser utilizado desde a admissão do paciente − inclusive naqueles que vinham em uso de clopidogrel pode haver troca por ticagrelor. Prasugrel deve ser administrado apenas nos casos de certeza de angioplastia em pacientes que não faziam uso anterior de clopidogrel. Quando houver somatória de diversos fatores de alto risco hemorrágico (muito idosos, mulheres, baixo peso, uso de varfarina e insuficiência renal), o clopidogrel é uma opção mais segura e viável.

Considerando os resultados dos estudos TRITON e PLATO, os inibidores da glicoproteína (GP) IIb/IIIa tornaram-se opções de segunda linha, que podem ser adicionados como terapêutica tripla, associada ao AAS e ao inibidor de ADP, em casos selecionados de pacientes com SCA submetidos à angioplastia (elevada carga trombótica, *no-reflow *e embolização distal).

## ANTICOAGULANTES

Anticoagulantes são drogas que inibem a geração de trombina e/ou sua atividade. A utilização de anticoagulantes em SCA é área de ativa investigação. É difícil obter conclusões definitivas sobre a melhor estratégia anticoagulante, devido a diferentes tempos de tratamento, incerteza sobre doses anticoagulantes equipotentes e diferentes drogas antiagregantes utilizadas nos estudos. Diversos anticoagulantes foram testados, mas, atualmente, quatro drogas estão disponíveis para uso em SCASS-ST: heparina não fracionada, enoxaparina, fondaparinux e bivalirudina (Quadro 2).

**Quadro 2:** Tratamento anticoagulante

Fármaco	Dose	Tempo de uso	Contraindicação	Efeitos colaterais	Ajustes	Observação
Heparina não fracionada	Ataque IV: 60UI/kg máximo 5.000UI	48 horas ou interromper após angioplastia	Sangramento ativo Trombocitopenia	Trombocitopenia Sangramento	De acordo com TTPA	Antídoto: protamina
	Manutenção IV:12UI/kg/h (máximo 1.000UI/h)			Aumento de transaminases		
	Manter TTPA entre 50 e 70 segundos (relação 1,5 a 2,0)					
	Na angioplastia manter TCA 200-300 segundos					

Enoxaparina	1mg/kg subcutâneo a cada 12 horas até 100kg	8 dias ou interromper após angioplastia	Sangramento ativo Trombocitopenia	Trombocitopenia Sangramento	50% redução dose em IRA/IRC	Monitorar anti-Xa em obesos, idosos, IRA/IRC
	Para angioplastia, se última dose entre 8-12 horas ou se recebeu somente uma dose de enoxaparina: 0,3mg/kg IV			Aumento de transaminases	Opcional: 25% redução em muito idosos	Antídoto: protamina (efeito parcial)

Fondaparinux	2,5mg subcutâneo 1 vez ao dia	8 dias ou interromper após angioplastia	Sangramento ativo	Sangramento, anemia	Sem ajustes	Associar heparina se angioplastia
			*Clearance* Creatinina <20mL/min			

IV: intravenoso; TTPA: tempo de tromboplastina parcial ativada; TCA: tempo de coagulação ativada; IRA: insuficiência renal aguda; IRC: insuficiência renal crônica.

### Heparina não fracionada

A heparina não fracionada (HNF) é uma mistura heterogênea de polissacarídeos com peso molecular variando entre 2.000 e 30.000 daltons. Age ligando-se à antitrombina e potencializa sua ação. Apresenta estreita faixa terapêutica e necessita de frequente monitorização por meio do tempo parcial de tromboplastina ativada.

A anticoagulação com HNF tem sido a pedra angular da terapia para os pacientes com AI/IAMSS-ST baseada em vários estudos randomizados que constataram taxas menores de morte ou reinfarto com a associação HNF e AAS, em relação ao AAS isoladamente.^22,23^

A administração de HNF deve ser iniciada com bólus endovenoso inicial de 60 unidades por kg até o máximo de 5.000UI, seguida por infusão contínua de 12UI/kg/hora até o máximo de 1.000UI/hora e ajustes por meio do tempo parcial de tromboplastina ativada, com alvo entre 50 e 70 segundos. Recomenda-se aferir diariamente as taxas de hemoglobina, hematócrito e plaquetas.

### Enoxaparina

As heparinas de baixo peso molecular (HBPM) são obtidas pela despolimerização das HNFs e a seleção daquelas com menores pesos moleculares (entre 2.000 e 10.000 daltons). Apresentam melhor absorção subcutânea, menor ligação a proteínas, menos ativação plaquetária e efeito mais previsível e reprodutível. Geralmente, não é necessário o controle da anticoagulação nem ajuste da dose. Apenas em pacientes com insuficiência renal ou, eventualmente, em obesos e idosos, recomenda-se o controle da ação da HBPM com mensuração da atividade anti-Xa.

Os estudos ESSENCE^24^ e TIMI 11B,^25^ que compararam enoxaparina com HNF associadas com dois antiplaquetários, em pacientes em estratégia conservadora inicial, sugeriram benefício anti-isquêmico com enoxaparina.

No estudo SYNERGY,^26^ que envolveu 10.027 pacientes que receberam estratégia de tratamento contemporânea, com a realização de angiografia precoce e o uso de inibidor de plaquetário da GP IIb/IIIa, enoxaparina e HNF, foram obtidos resultados anti-isquêmicos semelhantes. Houve mais sangramento com enoxaparina utilizando os critérios de TIMI (9,1% *versus* 7,6%; p=0,008), mas sem diferença estatisticamente significante utilizando os critérios GUSTO ou maior necessidade de transfusão. Aqueles que utilizaram uma formulação de heparina e, posteriormente, a outra antes da cineangiocoronariografia apresentaram taxas mais altas de sangramento e no desfecho composto de morte ou infarto.

Pacientes com AI/IAMSS-ST de alto risco, submetidos à estratégia invasiva precoce, inclusive aqueles em uso de inibidores da GP IIb/IIIa, podem receber enoxaparina ou HNF. No entanto, após a escolha de uma delas, recomenda-se a manutenção da mesma medicação até o final do tratamento.

### Fondaparinux

Fondaparinux é um pentassacarídeo sintético análogo ao sítio de ligação da antitrombina presente nas moléculas de heparina. Age neutralizando o fator Xa e, assim, previne a geração de trombina. Apresenta excelente biodisponibilidade após injeção subcutânea e meia-vida plasmática de 17 horas, o que possibilita sua administração uma vez ao dia. Droga de eliminação exclusivamente renal, não deve ser utilizada em pacientes com *clearance *<20mL/minuto. Nenhum caso definitivo de trombocitopenia autoimune induzido por fondaparinux foi relatado.^27^

No estudo OASIS-5,^28^ 20.078 pacientes com SCA sem supradesnivelamento de ST (SCASS-ST) foram randomizados para receber 2,5mg de fondaparinux subcutâneo uma vez ao dia, ou enoxaparina 1mg/kg duas vezes ao dia, por 8 dias ou até a alta hospitalar. O grupo que utilizou fondaparinux apresentou redução do risco de eventos isquêmicos semelhante ao grupo que fez uso de enoxaparina e ocorreu diminuição substancial das taxas sangramento maior (2,2 *versus *4,1%; p<0,001) e fatal (7 *versus *22%; p=0,005), respectivamente.

Fondaparinux esteve associado à menor mortalidade em 30 dias (2,9% *versus *3,5%; p=0,02) e 180 dias (5,8% *versus *6,5%; p=0,05). Porém, nos pacientes que realizaram revascularização percutânea, ocorreu maior trombose relacionada ao cateter no grupo fondaparinux (0,9% *versus *0,4%; p=0,001), o que resultou na recomendação do uso de HNF ou bivalirudina nos pacientes em uso de fondaparinux, que são submetidos à angioplastia. Apesar desse excelente resultado com a utilização de fondaparinux, é necessário citar que, no grupo enoxaparina, muitos pacientes receberam dose adicional de HNF no momento da angioplastia, prática atualmente contraindicada, devido ao aumento dos riscos de sangramento.^26^

Em suma, fondaparinux se mostrou uma opção mais segura para pacientes com SCA. Os pacientes com SCASS-ST em tratamento conservador se beneficiam com menor risco de sangramento e, se indicada angioplastia, o acréscimo de HNF durante o procedimento previne a trombose associada ao cateter, sem aparentemente incrementar o risco hemorrágico. Representa uma alternativa em estratégia invasiva (<72 horas após a admissão). Naqueles que necessitam de procedimento invasivo de urgência/emergência (<2 horas após a admissão), fondaparinux não é recomendado e, provavelmente, HNF ou bivalirudina são as melhores drogas nesta situação.^27^

### Bivalirudina

Bivalirudina pertence ao grupo dos inibidores diretos da trombina, que se ligam e inativam um ou mais locais da molécula de trombina. Bivalirudina é um polipeptídeo sintético análogo à hirudina. Como não se liga às proteínas plasmáticas, seu efeito anticoagulante é mais previsível. Ao contrário das heparinas, não necessita de cofator para agir e pode inibir a trombina ligada ao coágulo. A meia-vida é de aproximadamente 25 minutos em pacientes com função renal normal, e os parâmetros da coagulação retornam ao normal aproximadamente 1 hora após sua interrupção.

Bivalirudina foi testada em pacientes com SCASS-ST no estudo ACUITY.^29^ Foram randomizados 13.819 pacientes em três grupos: HNF ou enoxaparina associada com inibidor da GP IIb/IIIa; bivalirudina associada a inibidor da GP IIb/IIIa; ou somente bivalirudina (9,1% dos pacientes desse último grupo receberam inibidor da GP IIb/IIIa). Os desfechos compostos de isquemia em 30 dias (morte de qualquer etiologia, infarto do miocárdio ou revascularização não planejada) foram semelhantes nos três grupos, mas ocorreu menor taxa de sangramento maior no grupo que utilizou apenas bivalirudina em comparação com heparinas + inibidor da GP IIb/IIIa (3,0% *versus *5,7%; p<0,001).

É possível afirmar que bivalirudina, em associação com antiagregação dupla (ou tripla em casos selecionados), tem o mesmo perfil de eficácia e menores índices de sangramento que heparina em antiagregação tripla utilizada de forma rotineira e precoce, mas não temos dados para simplesmente afirmar que bivalirudina é superior às heparinas em SCA. Não recomendamos antiagregação tripla de forma rotineira nos pacientes com SCASS-ST.

A escolha da associação antiagregante e anticoagulante envolve características dos pacientes, risco de sangramento, disponibilidade de drogas e definição de estratégia invasiva ou conservadora. A figura 1 sugere um fluxograma de tratamento antitrombótico conforme estratificação, nas SCASS-ST no Hospital Israelita Albert Einstein.

**Figura 1:** Fluxograma de tratamento antitrombótico conforme estratificação, nas síndrome coronária aguda sem elevação de ST do Hospital Israelita Albert Einstein

## ESTRATÉGIAS CONSERVADORA E INVASIVA

Após o diagnóstico de SCASS-ST, além do tratamento farmacológico, os pacientes devem ser submetidos a algum tipo de estratégia de estratificação com exames subsidiários para avaliação coronariana funcional (testes de isquemia não invasivos) ou anatômica (cineangiocoronariografia). De maneira geral, a escolha do método depende do risco do paciente, da presença de comorbidades, da expectativa de vida, do *status* funcional e da disponibilidade dos métodos de estratificação de cada serviço médico. Podemos optar por: cineangiocoronariografia imediata (nas 2 horas seguintes), estratégia invasiva (cineangiocoronariografia dentro de 48 a 72 horas) e estratégia conservadora.

Recomenda-se cineangiocoronariografia imediata em SCASS-ST para os seguintes grupos de pacientes instáveis que apresentam alto risco de evolução desfavorável: angina recorrente ou persistente, apesar de tratamento clínico intensivo; instabilidade hemodinâmica; disfunção ventricular severa e insuficiência cardíaca; arritmia ventricular sustentada; e complicações mecânicas (insuficiência mitral aguda e defeito do septo ventricular).

A estratégia invasiva (cineangiocoronariografia com a intenção de revascularização dentro de 48 a 72) pode limitar a extensão do infarto e melhorar o prognóstico em pacientes SCASS-ST de moderado e alto risco.^30^ Sugere-se optar pela estratégia invasiva quando existirem as seguintes características, sempre considerando o risco hemorrágico e o *status* funcional dos pacientes: escore de TIMI ≥3; escore de GRACE ≥108; elevação de troponinas; infradesnivelamento do ST novo ou presumivelmente novo; fração de ejeção <40%; angioplastia coronária nos últimos 6 meses ou cirurgia de revascularização do miocárdio prévia; e angina pós-infarto.

Nos pacientes de mais alto risco, como aqueles com escore de GRACE ≥140, a estratégia invasiva dentro das primeiras 24 horas (14 horas em média) foi superior no estudo TIMACS (*Timing of Intervention in Acute Coronary Syndrome*)^31^ quando comparado à estratégia invasiva após 36 horas (50 horas em média).

A estratégia conservadora consiste na realização de teste de isquemia não invasivo após o evento. Teste ergométrico, cintilografia do miocárdio com estresse farmacológico (adenosina, dipiridamole dobutamina) ou exercício, e ecocardiograma com dobutamina. Qualquer um dos métodos disponíveis tem sensibilidade para detecção de isquemia e avaliação do risco do paciente. Opta-se pelo mais adequado, de acordo com a disponibilidade do serviço médico, condições físicas do paciente (capacidade de se exercitar), tolerância a fármacos (asmáticos não devem utilizar adenosina ou dipiridamol) e eletrocardiograma de base (pacientes com bloqueios de ramo, marca-passo ou sobrecarga ventricular acentuada devem submeter-se a exames de imagem).

Nos pacientes de baixo risco (escore de GRACE ≤108 ou TIMI ≤2) e que não apresentem nenhuma das características descritas previamente que indicam benefício com a utilização de estratégia invasiva, a opção inicialmente conservadora parece ser a mais adequada.

## CONCLUSÃO

O tratamento das síndromes coronárias agudas sem supradesnivelamento do ST está em contínua evolução, com a inclusão de novas drogas antiagregantes e anticoagulantes, que objetivam maior eficácia anti-isquêmica e menores índices de sangramento. A correta abordagem terapêutica, com a utilização de intervenções baseadas em evidências, associada com medidas de prevenção realmente efetivas, pode ajudar a diminuir a morbimortalidade.
